# A parallel English-Akan maternal health dataset to support machine translation and text-to-speech systems

**DOI:** 10.1016/j.dib.2026.113088

**Published:** 2026-07-18

**Authors:** Isaac Wiafe, Akon Obu Ekpezu, Fiifi Baffoe Payin Winful, Akosua Nyarkoa Wiafe-Akenten, Mark Atta Mensah, Nissi Semanyoh, Justice Kwame Appati, Sumaya Ahmed Salihs, Evans Kwasi, Gifty Odame, Kelvin Nketia-Achiampong, Kwaku Owusu Osei, Frank Ernest Yeboah

**Affiliations:** a Department of Computer Science, University of Ghana, Ghana; b Department of Computer Science and Information Systems, Ghana Institute of Management and Public Administration, Ghana; c University Health Services, University of Ghana, Ghana

**Keywords:** Akan, Low-resource languages, Maternal health, Machine translation, Natural language processing, Parallel corpus, Pre- and post-natal care, Text-to-speech

## Abstract

Audio and text datasets are essential for developing machine translation, text-to-speech systems, and speech-enabled systems. However, domain-specific datasets for low-resource African languages remain limited, particularly in the healthcare domain. This study addresses this gap by introducing a parallel English–Akan maternal health dataset to support machine translation and text-to-speech systems. The dataset consists of 3487 unique English maternal health phrases (question-and-answer) generated from verified digital health sources and medically reviewed for Ghanaian contextual relevance. These phrases were translated into Akan by four linguistic experts, producing 12,000 Akan transcriptions and 12,000 corresponding Akan audio recordings, totalling 32.313 h. The dataset covers prenatal and postnatal maternal health themes that are aligned with the WHO-recommended domains. The audio recordings were captured in soundproof vocal booths and processed into .wav format. This dataset provides a valuable resource for advancing Akan health communication technologies, maternal health chatbots, and low-resource language AI research.

Specifications Table

**Table:** 

Subject	Computer Sciences
Specific subject area	Maternal health machine translation and text-to-speech systems for low-resource languages
Type of data	Text (parallel English-Akan Question and Answer text corpus with metadata) Audio recordings of Akan text
Data collection	English maternal health content was scraped from verified international and Ghanaian digital-health websites. The OpenAI API (ChatGPT 4o) was used to generate 20,906 question-answer pairs using the scraped content. These pairs were reviewed and validated by a Ghanaian medical expert for their contextual relevance and medical appropriateness. After review and separation of the questions and answers into individual records, the final dataset contained 3487 unique English phrases. Four Akan linguistic experts translated the validated phrases into Akan using a web-based application. They provided one original translation and up to three alternative paraphrases for each phrase. They then read the transcribed texts in a soundproof recording booth. This resulted in 12,000 Akan text and audio sentences (equivalent to 32.313 h of audio) of maternal health-related question-and-answer content. Gadgets used: two soundproof vocal booths, 4 Samsung galaxy tablets, i5 and M2 MacBooks, audio-Technica microphones and headphones, scarlett 2i2 studio, 2 microphone stands, pop filters, 2 shock mounts, 2 dog clicks Software: Adobe Audition
Data source location	Country: Ghana Region: Eastern Region Community: Kwahu Source material: Verified digital health websites, both international and local to Ghana
Data accessibility	Repository name: Science DataBank Data identification number: https://doi.org/10.57760/sciencedb.32698 Direct URL to data: https://www.scidb.cn/en/detail?dataSetId=c633d8fdee6447a28c2ae2ea33ab2d73 [1]
Related research article	-

## 1 Value of the Data

• This dataset provides a domain-specific maternal health corpus for Akan, a low-resource Ghanaian language with limited publicly available digital health data. The dataset combines medically reviewed content, Akan translations, alternative paraphrases, and aligned speech recordings, making it relevant to both language technology and digital health research fields.
• The dataset supports research at the intersection of natural language processing, speech technology, machine translation, health informatics, and low-resource language AI. Its structure allows researchers to study English–Akan translation, Akan linguistic variation, maternal health terminology, text-to-speech and speech-to-text alignment in a medically relevant context.
• Researchers can use the text pairs to train, fine-tune, or evaluate machine translation models, particularly for English-to-Akan and Akan-to-English translations in the health domain. Alternative paraphrases can also support research on semantic variation, paraphrase generation, and robust language understanding in Akan.
• The Akan audio sentences can be used to develop or evaluate automatic speech recognition, text-to-speech, and voice-enabled conversational systems. Because the audio corresponds to translated maternal health question–answer content, the dataset can support multimodal research involving both spoken and written forms of Akan.
• The dataset can support the development of maternal health chatbots, voice assistants, health information retrieval systems, and local language health communication tools for Akan-speaking users. The expert review and contextual adaptation make the data suitable for studies requiring medically appropriate and Ghanaian-context maternal health content.
• The domain-specific focus on prenatal and postnatal maternal healthcare, with thematic categories aligned with the World Health Organization (WHO) recommended maternal health domains, makes this dataset particularly valuable for building health communication tools that can improve maternal health outcomes in Akan-speaking populations by reducing language barriers.
• The audio filenames follow a consistent naming convention that links each audio file to its corresponding record in the metadata file, thereby supporting traceability, automated playback, and integration into speech- and language-technology workflows.

## 2 Background

African languages remain underrepresented in natural language processing (NLP), machine translation (MT), and speech technologies, particularly in specialized domains such as health care. Compared with high-resource languages, there is a shortage of large, high-quality, and domain-specific datasets for African languages. These limitations hinder the development of language technologies and contribute to challenges in domain adaptation and performance of AI models in healthcare contexts [2].

Although Akan is a widely spoken language in Ghana, existing parallel corpus efforts are limited and multi-domain rather than tailored for clinical use [3]. This limitation has significant implications for maternal health communication, where low health literacy and language barriers can affect access to health information and contribute to poor maternal and fetal outcomes. Most expectant mothers, particularly in rural Ghana, have poor knowledge of antenatal care and are not functionally literate [4]. Yet, tailored interventions for linguistically diverse pregnant women are not common [5].

To address this gap, this study generated an English-Akan parallel corpus focused on maternal healthcare. The dataset is aligned with the World Health Organization (WHO) maternal health domains and is intended to support machine translation, text-to-speech technologies, maternal health chatbot development, and broader healthcare NLP research for low-resource African languages.

## 3 Data Description

The Akan maternal health dataset is based on 3487 unique English maternal health question-and-answer phrases that were translated into Akan and expanded with alternative Akan paraphrases. Of these 3487 unique English phrases, 3437 (98.566%) had more than one Akan variant, resulting in 11,950 Akan transcriptions. The remaining 50 phrases (1.434%) each have a single Akan variant. Altogether, the dataset consists of 3487 unique English maternal health phrases and 12,000 Akan transcriptions corresponding to 32.313 h of Akan audio recordings. The overall Akan maternal health dataset size is approximately 21.09 GB and the audio files ranges from 2.08 s to 42.71 s.

The 12,000 transcriptions and audio recordings were produced by four Akan linguistic experts (three females and one male). Each expert translated, transcribed, and recorded 3000 Akan sentences covering various maternal health thematic areas. Each row in the dataset represents either a question or an answer related to maternal health. Table 1 presents a detailed breakdown of the thematic distribution of records by linguistic experts.

**Table 1:** Detailed distribution of records across thematic categories by transcriber.

	Thematic category	BT		PT		IM		HA		Total
		Eng	Akan	Eng	Akan	Eng	Akan	Eng	Akan	
Prenatal	Physical Exercise	399	1378	0	0	0	0	36	108	1486
	Barriers and Myths	0	0	344	1158	0	0	0	0	1158
	Mental Health	0	0	307	1109	0	0	0	0	1109
	Childbirth Preparation	0	0	0	0	273	1087	0	0	1087
	Required Investigations	0	0	0	0	230	915	30	90	1005
	Medication and Vaccination	0	0	0	0	214	853	0	0	853
	Nutrition and Dietary Guidelines	0	0	47	187	3	7	294	816	1010
	Lifestyle and Health Behaviors	0	0	57	151	0	0	0	0	151
	Personal Hygiene	0	0	8	31	0	0	40	119	150
	Interventions for Common Diseases	6	18	0	0	0	0	0	0	18
Postnatal	Hygiene	231	788	0	0	0	0	99	393	1181
	Preventive Medicine	217	681	0	0	6	18	49	139	838
	Interventions for Complications	20	67	41	184	30	120	298	878	1249
	Mental Health	22	68	0	0	0	0	0	0	68
	Nutrition and Exercise	0	0	48	180	0	0	45	179	359
	Barriers and Myths	0	0	0	0	0	0	91	273	273
	Required Investigations	0	0	0	0	0	0	2	5	5
	**Total number of phrases**	**895**	**3000**	**852**	**3000**	**756**	**3000**	**984**	**3000**	**12,000**
	**Total number of audios (hours)**	**-**	**8.916**	**-**	**7.821**	**-**	**8.179**	**-**	**7.397**	**32.313**

BT, PT, HA (females), IM (male) represent the initials of the four linguistic experts.

**Fig. 1:**
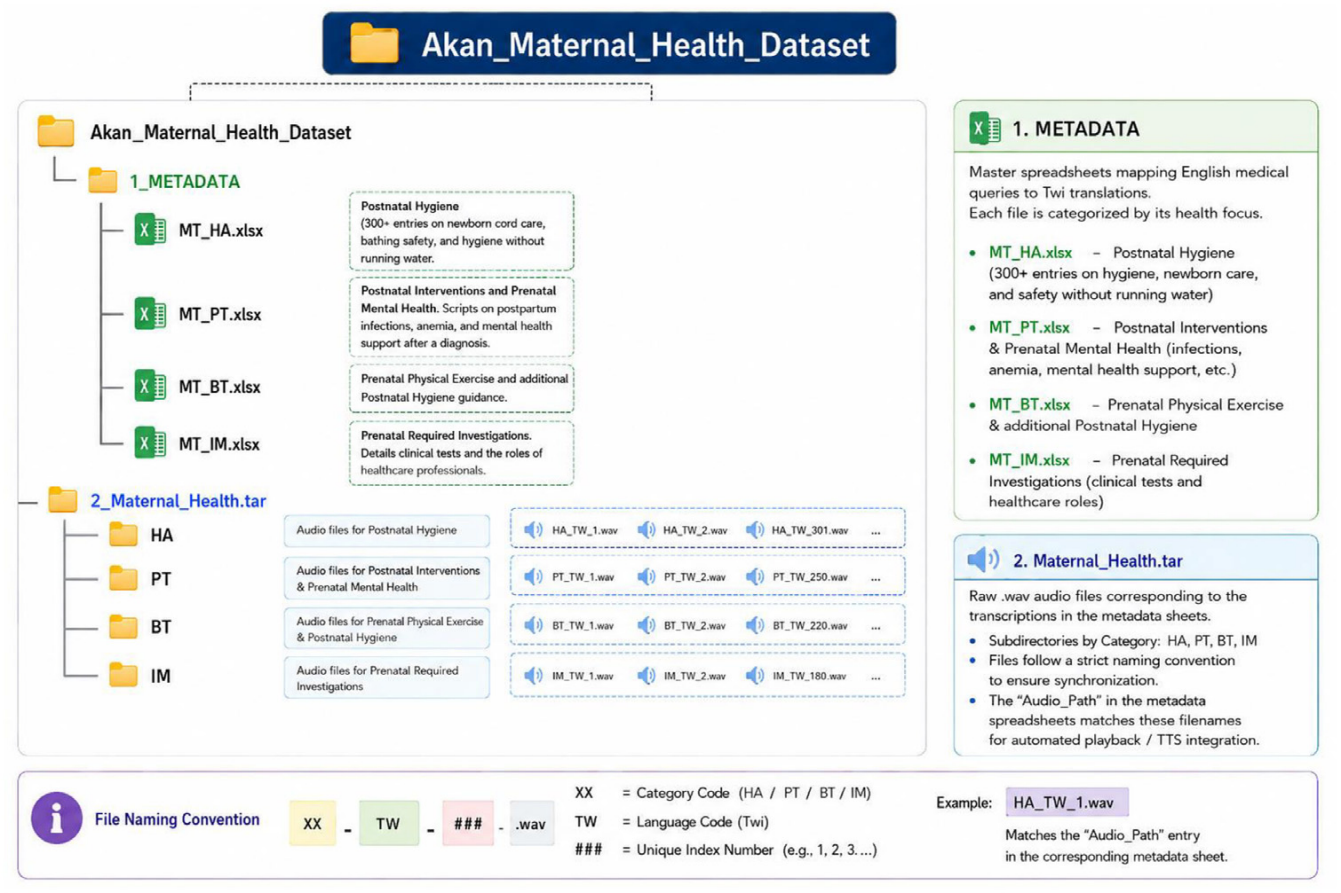
Visual structure of the Akan maternal health dataset.

Fig. 1 presents the structure of the Akan maternal-health dataset. The dataset consists of two main folders: a *METADATA* folder and an audio folder named *MT_Maternal_Health.tar*. The *METADATA* folder consists of four Excel files, each corresponding to translations by the four linguistic experts. Each Excel file contains 3000 records organized across five fields: ID (sequential integer, 1–3000), Phrase (English source text), Transcription (Akan translation), Theme (thematic category label), and Audio path. Table 2 summarizes the data fields in the *METADATA* folder.

**Table 2:** Description of metadata fields.

Field	Description
ID	Sequential unique identifier (1 to 3000)
Phrase	English source phrase (question or answer) related to maternal healthcare, generated via ChatGPT 4o from the scraped medical content.
Transcription	Akan translation or alternative paraphrase provided by Akan linguistic experts.
Theme	The thematic category labels were aligned with the WHO-recommended maternal health domains. The categories cover both prenatal and postnatal maternal health themes. The prenatal themes include Physical Exercise, Barriers and Myths, Mental Health, Childbirth Preparation, Required Investigations Medication and Vaccination, Nutrition and Dietary Guidelines, Lifestyle and Health Behaviors, Personal Hygiene, and Interventions for Common Diseases. The postnatal themes include Hygiene, Preventive Medicine, Interventions for Complications, Mental Health, Nutrition and Exercise, Barriers and Myths, and Required Investigations. Table 1 summarizes the distribution of records across these categories.
Audio path	The audio files are organized in a nested directory structure under the root folder *MT_Maternal_Health*, with XX as the sub-directory corresponding to the initials of the linguistic expert. The full directory path is therefore MT_Maternal_Health\XX\. All audio filenames follow a consistent naming convention using the prefix XX_TW_ and the file extension .wav. TW is the abbreviation for the dialect (TWI). The numeric component of each filename corresponds directly to the unique identifier in Column A. Thus, the filename pattern is XX_TW_{ID}.wav.

The *MT_Maternal_Health.tar* folder contains the corresponding Akan audio recordings. The audio files are organized into four subfolders, each containing recordings produced by one linguistic expert. The filenames follow a consistent naming convention that links each audio to its corresponding record in the metadata file

## 4 Experimental Design, Materials and Methods

The dataset was developed using a multi-stage methodology designed to ensure linguistic, clinical, and contextual relevance for maternal health in Ghana. We combined source text collection, AI-assisted content generation, expert review, human translation, audio recording, and post-processing. Akan linguistic experts were selected using purposive sampling methods. The expert group included three females and one male, each with a minimum academic qualification of MPhil in Akan and Linguistics.

### 4.1 Source text collection and content generation

English-language maternal healthcare content was scraped from verified digital health websites, both international and local to Ghana [[6], [7], [8]]. These sources provided textual data related to prenatal and postnatal care. A prompt was designed to use ChatGPT 4o, a Large Language Model (LLM), to extract relevant question-answer pairs from each of the scraped excerpts. The OpenAI API was used to generate question-answer pairs in batches, with batch sizes between 50 and 100 pairs per round. Each generated data pair was categorized according to the World Health Organization (WHO)-recommended maternal health domains (Table 1).

Prior to expert validation, 20,906 question-answer pairs were initially generated. Owing to data annotation constraints, only 8563 pairs underwent expert review. To ensure contextual relevance and medical accuracy, each generated question-answer pair was manually reviewed by a medical expert who is also a member of the research team. Following expert validation, the reviewed question-answer pairs were separated into individual questions and answers. This resulted in 3487 unique English maternal health phrases, which formed the basis of the dataset and were subsequently translated into Akan.

### 4.2 Human translation/transcription via a web application

**Fig. 2:**
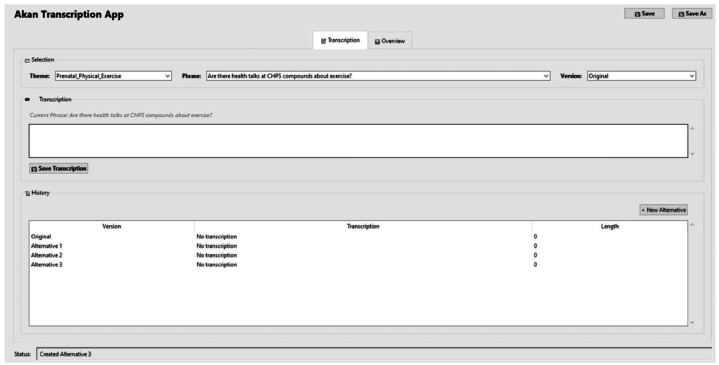
Screenshot of Akan transcription app.

To facilitate a structured method for collecting translation pairs, a web application (titled “Akan Transcription App”) was designed. The Akan Transcription App presented each English phrase (either a question or an answer) to the linguistic experts with selection fields for the thematic category (Theme), source phrase (Phrase), and version type (Original, Alternative 1, Alternative 2, or Alternative 3). The interface included a transcription input area, a “Save Transcription” button, and a history panel displaying all versions with their transcription texts and character lengths. Refer to Fig. 2 for a sreenshot of the transcription app.

The 3487 English question-and-answer phrases were shared among the Akan linguistic experts in batches of 200 pairs at a time until all 3487 phrases were exhausted. The experts were required to complete each assigned batch before requesting another. To capture the contextual nuances and linguistic variations of the phrases in Akan, the experts were asked to translate each question or answer into up to four Akan variants, consisting of one original translation and up to three alternative paraphrases. After the translation/transcription process was completed, the transcripts were exported to Excel, resulting in four files corresponding to the four linguistic experts. Each expert produced 3000 Akan transcribed texts, adding up to 12,000 Akan transcriptions. This stage was followed by transcription validation.

### 4.3 Transcription validation

The transcripts were validated to ensure linguistic accuracy, contextual appropriateness, orthographic consistency, and semantic fidelity of the Akan translations. After the initial translation/transcription process, the four exported files were subjected to independent validation involving proofreading and normalization. To reduce bias, the transcribed files were blindly reassigned among the linguistic experts. Each expert was assigned transcribed files that they had not originally translated or transcribed. This ensured that the validation process was conducted independently and that each transcript was reviewed by a second Akan language expert.

At the proofreading stage, linguistic experts checked whether the Akan translations preserved the intended maternal health message, whether the alternative paraphrases were semantically appropriate, and whether the phrasing sounded natural in Akan. Where multiple Akan variants were provided for the same English phrase, the experts assessed whether each variant was a legitimate alternative, rather than a repetition or distorted translation.

The experts also performed spell-checking and orthographic reviews. This included checking the correct use of Akan special characters, tone-related orthographic conventions where applicable, punctuation, spacing, capitalization, and consistency in the spelling of key maternal health terms. In cases where specific medical terms did not have direct Akan translations or where translation could distort the intended clinical meaning of the English word, the linguistic experts agreed on a consistent transliteration strategy that was contextually appropriate. For example, terms such as “cord stitching” were rendered by spelling the English term as it would naturally be pronounced in Akan. This approach preserved the clinical meaning of the original term while making the expression intelligible and pronounceable by Akan speakers. The reviewers also checked the alignment between the English phrase, Akan transcription, thematic category, and audio path to ensure that each record was complete and correctly structured.

The normalization stage involved harmonizing the transcripts to ensure consistency across all four folders. This was done by two Akan-speaking members of the research team and included standardizing repeated terms, correcting typographical errors, resolving inconsistencies in the use of Akan characters, and ensuring that similar medical concepts were expressed consistently across the dataset.

### 4.4 Studio setup and audio recording

Two soundproof vocal booths were set up in separate enclosed rooms. One booth used a digital mic (Audio Technica) that was connected directly to an M2 laptop, while the other booth used an analog Scarlett 2i2 Studio microphone that was connected to an i5 laptop through a sound card interface.

Each vocal booth was supported by a dedicated recording team. For the two booths, four studio controllers and four signal persons were assigned. That is, two studio controllers and two signal persons attached to each booth. During each recording session, one studio controller and one signal person were actively on duty with the recorder, and the others provided rotation support as needed. Thus, each active session involved three key roles: recorder, studio controller, and signal person.

Similarly, the four linguistic experts were divided into two groups, with each group assigned to a designated vocal booth. Within each group, the experts alternated between recording and rehearsing the script. Each expert recorded for approximately 30 min while their counterpart rehearsed their script in a different room. After each 30-minute recording session, both the recording team and the linguistic expert took a break, and the counterpart entered the booth to record. This rotation continued until all the experts completed the audio recordings of their scripts.

The scripts were read using a Samsung tablet. The signal person and controller also had copies of the scripts and followed the script alongside the recorder. This was to ensure that no words were added or omitted during reading. Both the recorder and controller wore headphones. The recorder’s headphones allowed the recorder to hear themselves, while the controller’s headphones allowed him to hear the recorder in real time.

At the beginning of each recording session, the controller created a new multitrack session in Adobe Audition (enter the recorder’s initials and script number as the file name, set storage location, set sampling rate to 48 kHz, bits depth to 32 bits, and set track to stereo (for Scarlett mic) and mono (for Audio Technica)). The controller also added multiple tracks corresponding to the number of sentences. The sound level was between −19 and −25 LUFS.

The linguistic experts were provided with guidelines on the use of a dog clicker as an audio marker during the recording. The guidelines were as follows:

i. Upon receiving a signal from the signal person, click the dog clicker before beginning each sentence and click at the end of each sentence.
ii. Click the dog clicker if any mistakes are made while reading or if the recorder wants to catch some breathe. This enables the controller to effectively manage the recording console.
iii. Please feel free to skip any sentence that you are not comfortable reading (there was no such case).
iv. Please feel free to send a signal at any point during your recording session if you want to take a break.

### 4.5 Audio processing

The recordings were completed over six days from Sunday to Friday. All files were in ‘.wav’ format. At the end of the recording process, four folders were created; one for each linguistic expert. All clicks within the recordings were deleted. A metadata file was created using Excel. It provided details on the audio transcriptions, audio path, and file name.

Given that both mono (from audio technical mic) and stereo (from the analog mic-scarlett) audio files were found within the dataset, the next step involved converting all files to mono. To minimize data loss and avoid unnecessary compression during conversion, Pydub library version 0.25.1 was used. The conversion script was designed to operate on one folder at a time and provide a log for every event at each stage of the process. This allowed for the maintenance of a clear record of the conversion process and troubleshooting of any issues that arose.

Some files initially identified as mono files were not true mono files. Despite being labeled mono, these files still accessed two audio channels (stereo), with one channel muted. Thus, the script was modified to convert ‘the mono’ into actual mono files. This enabled a uniform audio format for the dataset.

## Limitations

This study is limited by the distribution of thematic categories and dialects. The distribution across the thematic categories was uneven: the largest category and prenatal topics overall accounted for 66.9% of records versus 33.1% for postnatal topics. Although all English outputs by ChatGPT-4o were reviewed and validated by a medical expert, some bias may remain, including source-related bias from the websites used, uneven representation of maternal health topics, and the tendency of large language models to produce phrasing that may not fully reflect naturally occurring health communications.

Furthermore, while different variants of the Akan language, such as Twi, Fante, and Akuapem, exist, the dataset generated in this study is Twi only. Consequently, its direct applicability to other Akan varieties may be limited without further adaptation, validation, or additional data collection.

## Ethics Statement

Ethical approval for this study (the Obaa Panin Project) was obtained from the Ethics Committee for Basic and Applied Sciences (ECBAS), University of Ghana (Ref. No: ECBAS 029/24-25). The four linguistic experts signed a project-specific participant service and data-use agreement before participating in the recording sessions. The agreement informed participants about the purpose of the voice recording exercise, collection and processing of speech/voice data, possible use of the recordings for speech technology development, data protection provisions, and their rights and obligations as participants. Signed agreements were obtained prior to recording and were retained as part of the project documentation.

## Credit Author Statement

**Isaac Wiafe:** Conceptualization, Methodology, Data collection, Writing - revisions, Project administration, Supervision, Investigation, Resources, Funding acquisition. **Akon Obu Ekpezu:** Conceptualization, Methodology, Data collection, Project administration, Investigation, Writing – original draft and revision. **Fiifi Baffoe Payin Winful; Akosua Nyarkoa Wiafe-Akenten:** Investigation, Data collection and management, management of data collection tool, writing, and revisions. **Mark Atta Mensah; Evans Kwasi:** Investigation, Data collection and management, management of data collection tool. **Justice Kwame Appati:** Investigation, Conceptualization, Data collection and management, Writing - revisions. **Nissi Semanyoh:** Investigation, Writing – original draft. **Sumaya Ahmed Salih:** Investigation, development, and management of data collection tools. **Gifty Odame, Kelvin Nketia-Achiampong, Kwaku Owusu Osei, Frank Ernest Yeboah:** Investigation, Conceptualization, Data collection and management.

## Data Availability

Science DataBankAkan–English Maternal Health Parallel Corpus for Machine Translation (Original data)
